# Messing with cancer therapy: how the melanoma phenotype predicts checkpoint inhibitor response

**DOI:** 10.1038/s41392-024-01785-5

**Published:** 2024-04-01

**Authors:** Svenja Meierjohann, Corine Bertolotto

**Affiliations:** 1 Institute of Pathology, University of Würzburg, Würzburg, Germany; 2 University Côte d’Azur, Inserm, Biology and Pathologies of melanocytes, team1, Equipe labellisée Ligue 2020, Centre Méditerranéen de Médecine Moléculaire, Nice, France

**Keywords:** Skin cancer, Cancer therapy

In a study published in *Cell*, Pozniak et al. explored the spectrum of the phenotypic cell states of human metastatic melanomas in their tumor microenvironment before and early during immune checkpoint inhibitor (ICI) therapy and identified an enrichment of mesenchymal-like (MES) state melanoma cells in non-responders to ICI.^1^ These findings offer valuable insights into the factors contributing to the refractoriness of certain melanoma patients to ICI, while also unveiling novel molecular targets that hold promise for improving their treatment outcomes.

Metastatic melanoma only accounts for 4% of cutaneous cancer, but is responsible for 80% of skin cancer-related deaths. Over the last decade, immunotherapy with blocking antibodies against the immune checkpoint proteins PD1 and CTLA4 has revolutionized the treatment of this aggressive disease, improving the median patient survival from 6 to 9 months to ~6 years.

However, lack of initial response to immunotherapy can be observed in about 40–65% of metastatic melanoma patients, and secondary resistance can also develop. Therefore, the main clinical challenge is to overcome or prevent resistance to increase the number of complete responders. Previous studies analyzing melanoma tissues from ICI responders (R) and non-responders (NR) already identified an interferon-γ signature (which includes antigen presentation genes) as well as tumor mutational burden as prognostic factors for ICI response.^2^

The work by Pozniak and colleagues now adds a significant dimension to existing studies by conducting a high-resolution analysis of the ecosystem of metastatic melanomas before treatment and after one cycle of immune therapy. By using combined single-cell RNAseq and spatial multi-omics methods, they revealed several phenotypic clusters, which overlapped with those previously identified by the same laboratory in a mouse model of melanoma. Importantly, ampleness of these cell states was not associated with a specific mutational status, but was likely driven by the tumor niche and intercellular communications. Two of these clusters—dedifferentiated “MES” and “antigen presentation” - differed significantly in melanomas from responders (R) and non-responders (NR). Responders showed a generally higher amount of cells with antigen-presenting phenotype, which had a tendency to be located in close proximity to immune cells. In contrast, MES melanoma cells were more abundant in NR, particularly in early-on ICI treatment lesions. Significantly, both antigen presentation and MES cell populations were predictive of response to ICI and possibly of patient survival.

As the MES phenotype is also associated with resistance to BRAF/MEK inhibitors, which comprise the second common therapy for melanoma patients, discovering drivers of the MES signature is also relevant for improving this therapy approach.

**Fig. 1:**
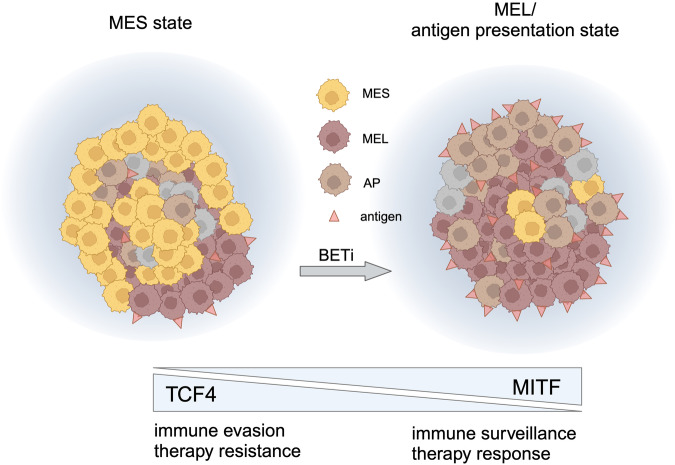
Schematic overview of the role of MES melanoma cells in non-responders. MES melanoma cells were found to be particularly enriched in melanomas from patients not responding to anti-PD1 or anti-PD-1 + anti-CTLA4 therapy. They express TCF4, which drives the MES signature, but blocks melanocytic (MEL) (MITF-driven and differentiated) and antigen presenting (AP) signature genes, thereby reducing immunogenicity. *TCF4* gene is bound by the chromatin reader protein BRD4. BET inhibition (BETi) can block TCF4 expression and reprogram melanoma cells to a less mesenchymal and more melanocytic and antigen-presenting phenotype. Please note that there are additional melanoma phenotypes within the tumors (represented by gray cells). Created with BioRender.com

Remarkably, the authors found that the basic helix-loop-helix transcription factor TCF4, a driver gene of the MES-related epithelial/mesenchymal transition (EMT) signature, was specifically expressed in the subtype of MES melanoma cells. Considering the effect of EMT genes in preclinical models of ICI resistance,^3^ the authors then further focused their attention on TCF4. They found TCF4 enrichment in their samples with MES signature. When the authors reduced TCF4 expression in MES cells, these adopted a differentiated and melanocytic (MEL) state, as illustrated by decreased levels of EMT genes and increased differentiation associated with expression of the melanocytic lineage marker MITF. Interestingly, TCF4 inhibition was also associated with an increased expression of genes involved in antigen presentation (AP) and IFN signaling (Fig. 1).

By compromising the differentiation (MEL) and antigen presentation programs, TCF4 therefore diminishes central determinants of the immune response and might be instrumental for the observed lack of therapy success in NR.

To functionally investigate this aspect, the authors used in vitro co-culture systems and mouse models. The data demonstrated that melanoma TCF4 impinged on T-cell killing activity in vitro. Accordingly, a *Tcf4* knockdown in melanomas improved the therapeutic effect of anti-PD1 antibodies in vivo.

Most importantly, although TCF4, as a transcription factor, is difficult to target with small molecules, the authors showed that a degrader of the bromodomain and extra-terminal domain (BET) protein BRD4 blunted TCF4 expression, compromised EMT, and enhanced differentiation and antigen presentation programs. In addition, both BET degrader as well as TCF4 knockdown sensitized melanoma cells to BRAF and MEK inhibitors in vitro. Therefore, BET inhibition mimicked TCF4 knockdown effects. Combinations of BET inhibitors with ICI will have to be studied in the future to test if this approach provides a therapeutic opportunity. In the melanoma population, TCF4 is specifically expressed in MES cells. Thus, instead of a systemic approach, antibody-drug conjugates exclusively targeting antigens expressed on the surface of MES cells may offer the possibility of directing BET degraders to this cell population and avoid adverse effects.

TCF4 is a known inducer of EMT in epithelial cancers. In melanoma, little is known about this transcription factor. However, studies in melanocytes revealed that TCF4 suppresses the transcription of the melanocytic lineage factor MITF.^4^ MITF plays a crucial role in shaping melanoma cell plasticity and heterogeneity, presenting significant challenges to successful immunotherapy. Moreover, MITF controls processing as well as the surface presentation of antigens and regulates the expression of coinhibitory receptors, including PD-L1. It furthermore affects the inflammatory secretome of melanoma cells, thus influencing the recruitment of immune cells and anti-tumor responses.^5^ Considering these aspects and the fact that TCF4 impaired MITF expression, TCF4 might exert its immune-modulatory effects, at least partly, through MITF.

In summary, overcoming drug resistance remains the primary obstacle to attaining cures for cancer patients. The research conducted by Pozniak and colleagues not only sheds light on the reasons behind melanoma patients’ resistance to ICI but also unveils novel molecular targets to improve outcomes. These could be relevant for additional tumor entities, such as breast cancer, in which mesenchymal cell states are commonly found.
